# The Efficacy of a Smartphone Game to Prevent HIV Among Young Africans: Protocol for a Randomized Controlled Trial in the Context of COVID-19

**DOI:** 10.2196/35117

**Published:** 2022-03-03

**Authors:** Victor Mudhune, Gaëlle Sabben, Ken Ondenge, Calvin Mbeda, Marissa Morales, Robert H Lyles, Judith Arego, Richard Ndivo, Robert A Bednarczyk, Kelli Komro, Kate Winskell

**Affiliations:** 1 HIV Research Division Center for Global Health Research Kenya Medical Research Institute Kisumu Kenya; 2 Hubert Department of Global Health Rollins School of Public Health Emory University Atlanta, GA United States; 3 Department of Biostatistics and Bioinformatics Rollins School of Public Health Emory University Atlanta, GA United States; 4 Department of Behavioral, Social, and Health Education Sciences Rollins School of Public Health Emory University Atlanta, GA United States

**Keywords:** HIV, young Africans, adolescent, Kenya, serious game, game for health, randomized controlled trial, mHealth, prevention, smartphone, teenager, young adult, Africa, gaming, COVID-19, efficacy

## Abstract

**Background:**

Adolescents contribute slightly less than one-third of all new HIV infections in sub-Saharan Africa. There is a need for more effective intervention approaches to help young adolescents safely navigate through adolescence and into adulthood. We are assessing the efficacy of *Tumaini*, a smartphone game designed to prevent HIV among young Africans. Against the background of COVID-19, meaningful alteration of the research protocol was necessary to ensure successful implementation and retention of the study participants in ongoing research.

**Objective:**

The objective of our protocol is to determine (1) if *Tumaini* delays sexual debut and increases condom use at first sex and (2) whether it influences behavioral mediators of early and unprotected sex.

**Methods:**

Participants were recruited from Kisumu County in Western Kenya. This study is a 2-arm, individual-randomized controlled trial that enrolled 1004 adolescents aged between 12 years and 15 years. The intervention arm participants are playing *Tumaini*, while the control arm is provided with *Brainilis*, a commercially available control game. The study period will last 45 months. At baseline, participants in both arms completed a baseline survey and biological testing for HIV and herpes simplex virus, type 2 (HSV-2); participants will have annual game play periods in years 1-3. They will also complete a total of 12 follow-up surveys. At endline, repeat biological testing will be conducted. Protocol adaptations were necessitated by the COVID-19 pandemic and implemented in accordance with local public health guidelines.

**Results:**

Participants were enrolled between October 2020 and November 2020. We plan to complete study procedures in September 2024. The enrolled participant sample was 50.1% (499/996) female and had a mean age of 14.0 (SD 0.6) years.

**Conclusions:**

This ongoing research demonstrates that, with appropriate revisions to planned protocol activities guided by the need to maintain study integrity, protect both study participants and staff, and adhere to institutional review board and local health authority guidelines, human subject research is possible in the context of a global pandemic. If the trial demonstrates efficacy, *Tumaini* would provide an alternative, remote means of delivering age-appropriate education to adolescents on safer sex, HIV prevention, and effective life skills on a highly scalable, low-cost, and culturally adaptable platform.

**Trial Registration:**

ClinicalTrials.gov NCT04437667; https://clinicaltrials.gov/ct2/show/NCT04437667

**International Registered Report Identifier (IRRID):**

DERR1-10.2196/35117

## Introduction

HIV prevention interventions need to be targeted and packaged to meet the needs of high-risk groups. Significant progress in the discovery and adoption of HIV prevention and treatment measures has resulted in a global decline in the incidence rate by 23% in the last 10 years [1]. To achieve the Joint United Nations Programme on HIV/AIDS (UNAID) Fast-Track strategy to end the AIDS epidemic by 2030 [2], countries need to use all the powerful tools available to them and a leave-no-one-behind approach. In sub-Saharan Africa, a region accounting for almost two-thirds of the global burden of new HIV infections, adolescent girls and young women account for 26% of new infections [3]. In Kenya, one-third of all new infections occur in young people aged 15 years to 24 years [4], with young women at particular risk.

Young people need comprehensive and correct knowledge and skills prior to sexual debut [5-7], to ensure they are able to protect themselves from HIV at their first sexual experience and onwards. Such knowledge and skills need to be well-packaged and age-appropriate. In sub-Saharan Africa, comprehensive HIV prevention knowledge among adolescents remains below 50% in most countries with available data [7-12]. Reaching adolescents with prerisk prevention interventions may help establish lifelong patterns of safer sexual behavior and avert high-risk behaviors in the future [13-16]. Interventions must promote skills for understanding and managing the risks of HIV infection that address important contextual drivers of adolescent risk and are relevant to local practices and culture [17-21]. Increasingly accessible smartphone technologies in Africa make it possible to engage youth—at scale and at low cost—in culturally adapted prevention interventions that require few resources to implement with consistent quality and motivational appeal and incorporate automated data collection [21-23]. If appropriately grounded in behavioral theory and evidence-based practice, electronic games delivered via mobile phones have the potential to become valuable tools in HIV prevention efforts.

*Tumaini* is a theoretically grounded, narrative-based game for inexpensive Android smartphones designed in collaboration with US-based and Kenyan specialists in adolescent sexual health, with input from Kenyan preadolescents and their parents. It aims to increase age and condom use at first sex by boosting knowledge about sexual health and HIV; building risk avoidance, risk-reduction skills, and related self-efficacy; challenging HIV stigma and harmful gender norms and attitudes; fostering future orientation, goal setting, and planning; and promoting dialogue with adult mentors [24,25]. *Tumaini* is grounded in (1) theory on narrative and narrative-based applied communication, (2) social behavioral theory and existing evidence-based HIV prevention interventions, and (3) principles of instructional design. Tumaini is made up of 3 integrated parts: (1) a central interactive narrative featuring 6 playable characters (3 male, 3 female) whom players guide into and through adolescence, making decisions that have short- and long-term consequences for the characters’ lives, relationships, and health; (2) a set of mini-games that tie into the narrative and support and reinforce its core themes; and (3) “My Story,” in which the player sets goals, plans how to achieve them, and reflects on how the game relates to his or her life and how he or she might apply the lessons learned to protect his or her future. In a feasibility study with 60 preadolescents conducted in Kenya [26], the intervention showed significant gains in sexual health–related knowledge and self-efficacy, behavioral intention for risk avoidance strategies and sexual risk communication, and overall increased scores on the behavioral survey measures when compared with the control arm at 6 weeks postintervention [27]. Intervention arm participants spent, on average, over 50% longer playing the game than instructed, while quantitative and qualitative data on user engagement and game appeal, relevance, and acceptability among adolescents and parents were extremely positive [28,29]. A randomized controlled trial (RCT), with a larger sample size (anticipated n=1000) and longer duration of follow-up (45 months), was proposed to evaluate behavioral efficacy of the intervention. A detailed protocol was developed that is the subject of this manuscript.

On January 30, 2020, the World Health Organization (WHO) Emergency Committee declared a global health emergency based on growing case notification rates of SARS-CoV-2, the cause of a respiratory illness designated “coronavirus disease 2019” or COVID-19 [30]. WHO published a comprehensive package of guidance documents for countries covering topics related to the management of the outbreak including infection prevention and control measures against COVID-19 [31,32]. On March 11, 2020, COVID-19 was declared a pandemic, and Kenya confirmed its first case on March 13, 2020 [33]. Various public health infection prevention measures were put in place across the country, including in Kisumu, the site of the study. These were expected to be implemented for an extended period and included wearing of masks in public spaces, a regional travel ban, limitations on public gatherings, night curfews, screenings for fever, massive handwashing campaigns, and closure of schools. Although some remote schooling was possible in certain cases (eg, private schools), for the most part, school-aged children remained out of school until October 2020. Research activities were modified to comply with at-the-time current guidance provided by the Kenyan Government and the institutional review boards (IRBs) overseeing study implementation.

This study, which began implementation in October 2020, is being carried out in Kisumu in the context of the COVID-19 pandemic and related public health disease containment measures. The purpose of this paper is to present the protocol for the efficacy RCT, with particular attention to adaptations from the previous pilot feasibility study, and to inform others about feasible and acceptable protocol modifications in the context of a respiratory virus pandemic.

## Methods

### RCT Design

This study is a 2-arm, non-blinded, individual RCT targeted to enroll 1000 adolescent participants aged 13 years to 14 years. The intervention-arm participants are playing *Tumaini*, an interactive, narrative-based electronic game, which is loaded on study-provided, low-cost Android smartphones. Adolescents in the control arm are receiving standard of care (ie, no intervention beyond any existing sex education from family, school, and peers) and allocated Android smartphones loaded with *Brainilis*, a free game that challenges the brain in areas like memory, logic, math, and focus, downloaded from Google Play Store. Selection of *Brainilis* as an attention-control game [34] was based on free download availability in Kenya, age-appropriate educational content, a lack of overlap with *Tumaini* in terms of content and game style, and perceived interest for the age group. The intervention period will last 45 months, with participants in both arms completing a baseline survey and 12 follow-up surveys, baseline and endline biological testing for HIV and herpes simplex virus, type 2 (HSV-2), game play periods during the first 3 years, and periodic qualitative data collection to monitor study acceptability and community concerns and to inform potential future dissemination. The 45-month study period is determined by the need for a high enough proportion of participants to become sexually active over the course of the study to allow us to determine the efficacy of the intervention.

The trial was preceded by cognitive interviews [35] for, and piloting and reliability testing of, the quantitative data collection instrument to be used during the trial. These activities were targeted to engage 200 adolescents aged 13 years to 14 years and 32 of their parents; they are henceforth referred to as “Phase 1” of this study.

### Ethics Approval

The IRB of Emory University (STUDY00002974) and Kenya Medical Research Institute (KEMRI) Scientific and Ethics Review Unit (SERU: KEMRI/SERU/CGHR/11/3812) approved this study.

### Implementation Setting

The study is being conducted in urban and peri-urban locations in East, West, and Central administrative locations of Kisumu County in Western Kenya. Kisumu is the country’s third largest city. Data collection is taking place at KEMRI facilities, health facilities, and community halls.

Against the background of the COVID-19 pandemic, study activities originally intended to be conducted in person were modified to comply, as needed in the context of an evolving epidemic, with current guidance provided by the Kenyan Government. This included guidance from the local Ministry of Health, Ministry of Education, and the Departments of Health and Education of the County Government of Kisumu. Emory IRB and the KEMRI SERU provided further guidance aiming to protect study staff and participants. In cases where these guidelines were not in accord, the most stringent guidance was followed.

### Target Population

The study recruited different sets of participants for the 2 phases of the study: survey revision and update activities (Phase 1). All participants had to be residents of Kisumu County and have had no previous exposure to or engagement in any research activities related to *Tumaini,* including the formative research.

#### Phase 1 Population

For this phase of the study, 200 adolescents and 32 parents of adolescents were recruited. Adolescents were required to have basic English literacy and Grade 3-4 on the Flesch-Kincaid Reading Scale (assessed via a short listening and reading comprehension test at enrollment) and were aged between 12 years and 14 years at recruitment. Parents and guardians for this phase had to be a parent or guardian (henceforth referred to as “parent”) to a child aged between 12 years and 17 years old at the time of recruitment. In addition to the aforementioned inclusion criteria, the possibility of remote data collection was necessitated by the COVID-19 situation for the activities related to updating the behavioral measures. Adolescent and parent participants were required to have access to an electronic device capable of running Zoom software [36] (ie, a tablet, smartphone, or computer).

#### Phase 2 Population

For the RCT, 1000 adolescents were to be recruited, as well as a subset of willing parents to the adolescents. In addition to the English language, lack of previous exposure to *Tumaini*-related activities (including survey revision under Phase 1), and age eligibility criteria, we limited enrollment to 1 child per family. In cases where multiple children in a family were eligible and interested, only the child whose name came first in the alphabet was enrolled.

A sample of the adolescent trial participants (n=16) allocated to the intervention arm of the study and a sample of their parents (n=12; including 6 parent-child dyads) were selected to participate in related and repeated annual qualitative research activities. Details of their selection are described in the following sections in the context of the qualitative activities themselves. At endline, 32 other intervention stakeholders, including health care workers, teachers, and community leaders, will supplement the qualitative sample. These additional stakeholders will be identified through stakeholder engagement and be required to have expertise on the sexual health needs of adolescents by virtue of their profession or status in the community to provide relevant insights.

### Recruitment and Enrollment

Prior to initiating recruitment activities, approval was obtained from local Ministry of Health and Ministry of Education officials within Kisumu County. Based on experience from the feasibility pilot study, this study had proposed to recruit adolescents through schools for both Phases 1 and 2, working with head teachers to distribute invitation letters to potentially eligible adolescents to deliver to parents or caregivers. Follow-up in-person informational meetings were then to be used to identify interested and eligible adolescents and parents for recruitment. Because schools closed due to COVID-19 restrictions, active school-based recruitment was not possible. Therefore, participant recruitment was initiated through a combination of strategies in order to ensure a diverse pool of potential participants and allow for more rapid recruitment.

For Phase 1 (survey revisions), the following strategies were employed: (1) schools distributing invitation letters to potentially eligible parents to inform them of the study using existing school communication channels, as well as sharing parents’ contact information after consulting those whose children were eligible based on school grade enrollment (grade 7 was identified as the target grade for ease of identification of potential participants as corresponding most closely to the desired age range); (2) recruitment directly in the community with support from the community advisory board (CAB) members’ ongoing activities; and (3) recruitment through parents or participants from previous studies who had consented to future contact and their social networks, employing a snowball approach.

For Phase 2 (the RCT), only strategies (1) and (2) implemented in Phase 1 were employed. The need for participants in this phase to be intervention-naïve, as well as concerns about the potential for contamination between study arms, in particular control-arm adolescents accessing the intervention through pre-existing social networks, precluded the use of snowball sampling from previous participants.

Multiple community meetings were held with the support of CAB members at different times in each area of the city to maximize accessibility to interested parents. KEMRI study staff made study presentations, with parents being invited to volunteer for participation. Community gatherings for recruitment and participant screening were conducted outdoors following pandemic guidance regarding number of participants, social distancing, and use of face coverings.

Following a phone discussion or in-person meetings maintaining social distancing, parents’ willingness to volunteer for participation was assessed, and interested participants were screened for eligibility based on adolescent age, grade level, and absence of previous participation in related study activities. Child’s age eligibility was confirmed via presentation of any official document giving the child’s name and date of birth (eg, birth certificate, baptismal certificate, or passport). Interested and eligible parents provided locator information, including a phone number and physical address, where they could be reached for future consent and assent procedures.

Recruitment slots were generated for the target 1000 adolescents for Phase 2, to ensure more targeted recruitment efforts and appropriate distribution of the sample by gender, age, and location (by geographic subzones within East, West, and Central Kisumu). Recruitment slots were filled as soon as a participant matching the slot characteristics was identified and confirmed willing to participate in the study. Priority was given to adolescents aged 13 years or 14 years old, and the sample was supplemented by 12-year-olds when necessary to achieve enrollment targets. Recruitment materials and consent and assent forms were available in Kenya’s national languages (English and Kiswahili) and most common local language within the catchment area (Dholuo) to ensure comprehension and were updated to provide for the possibility of remote consenting via phone or Zoom software, depending on participant’s preference and local government guidance. No participant opted for remote consenting, so all consenting was done in person, observing infection prevention measures.

### Survey Instrument

The behavioral survey instrument used in this study was adapted from the version used in the feasibility study, which is described elsewhere [27]. The survey was revised to remove or replace items that saw a ceiling effect, add items to assess social desirability bias (drawing from the Marlowe-Crowne scale adapted by Vu et al for use in Kenya [37]), and reinforce measurement of certain behavioral constructs to strengthen mediation analysis. In addition, measures were added that had not been appropriate for inclusion in the feasibility study but were warranted for this study because of its length, longitudinal nature, and older participants. Examples include measures related to sexual risk (number of partners, age differential with partners, marriage and pregnancy history, substance use) borrowed from the Project AIM evaluation conducted with adolescents of a similar age in Botswana [38]. Participants are also completing an additional set of game experience survey items. This is included as a component of the behavioral survey or administered independently after each game play period. This questionnaire was updated from that used during the feasibility study [27,29] to include psychological process measures, such as intrinsic motivation, immersion, and identification, as well as items focusing on communication about the game with peers and members of their households, especially parents, and questions to assess potential contamination across study arms.

As a proxy for socioeconomic status (SES), 2 questions were added regarding food insecurity [39] and 2 about house (walls and roof) building materials, drawn from the survey questionnaire used in the routine KEMRI/Centers for Disease Control and Prevention Health and Demographic Surveillance System [40] in Western Kenya. During survey piloting and reliability testing, there was little variation in responses to the roof materials question; hence, it was removed. The final survey instrument includes 109 questions. No single participant will be exposed to the full 109 items at any one time, due to a combination of skip patterns based on participant gender (eg, “Have you started your menses?”), gate questions (eg, “Have you ever had sexual intercourse, or sex?”), and certain questions only being asked at specific time points (eg, social desirability questions will be annual; experience of pregnancy will only be asked at endline).

The survey is delivered via tablet-based Open Data Kit (ODK) software [41] and includes an audio component to ensure consistent understanding of the questions among those with more limited English literacy. This delivery platform was a modification from the feasibility study that used an audio computer-assisted self-interview (ACASI) system. The change of platform allowed the team flexibility in conducting the surveys outside the KEMRI offices on multiple tablets at no additional cost, an essential criterion given the number of participants and tight period for data collection during school holidays. This approach accommodated the additional complications related to social distancing and in-person meeting limitations during pandemic-era implementation.

### Survey Revision

Parents of children similar to those to be included in the RCT were invited to review the full survey in focus group discussions (FGDs) to ensure its acceptability to parents (women, n=8; men, n=7). Subsequently, the survey underwent cognitive interviewing with adolescents to ensure cultural, linguistic, and age appropriateness, with particular attention paid to new questions as detailed in the previous section. We planned 5 rounds of 4 cognitive interviews, but, due to time constraints, we conducted 13 interviews with adolescents (girls, n=6; boys, n=7) to ensure acceptability, face validity, and comprehension, with revisions after each round. Both the survey and the proposed ODK platform were tested for acceptability, by piloting the final instrument complete with audio of all the questions through 2 rounds with 15 adolescents aged 12 years to 14 years using the tablets and headsets intended for study use. Each pilot participant was debriefed by study staff to identify any remaining barriers to comprehension in the survey or issues navigating the survey interface on the tablet. The survey and instructions were updated between piloting rounds, drawing on this feedback.

Assessment of reliability using test-retest was conducted with 150 adolescents aged 12 years to 14 years who took the survey twice approximately 1 month apart using the ODK tablet platform with audio. This sample size represented a balance between budgetary feasibility and minimum acceptable sample size for assessing questionnaire reliability. Temporal reliability of the survey items using Cohen kappa and Goodman and Kruskal gamma coefficients (in SAS/STAT© software, version 9.4) was calculated for participants’ answers over the 2 time points. For items where both coefficient values fell below the recommended threshold for acceptable reliability (typically 0.5) [42], the importance of the item to the overall goals of the study was reviewed to ensure that all thematic and theoretical areas of interest were appropriately assessed. In rare cases where an item was deemed too important to remove from the questionnaire, advice was sought from study staff and survey design experts on the team on strategies to boost reliability, and the item was revised as appropriate.

### Randomization

Participants were randomized 1:1 to either the control group (*Brainilis*) or the intervention group (*Tumaini*) at a time point between enrollment (consenting) and the baseline survey. Individual randomization was revealed to adolescents after they had completed all baseline data collection activities. The assignments were generated using PROC PLAN in SAS version 9.4 [43,44] to create gender-stratified randomization codes using block randomization with a block size of 10. Other demographics (age, geographic location, school) were expected to be balanced due to the slot allocation process used during recruitment.

### Study Procedures

**Figure 1:**
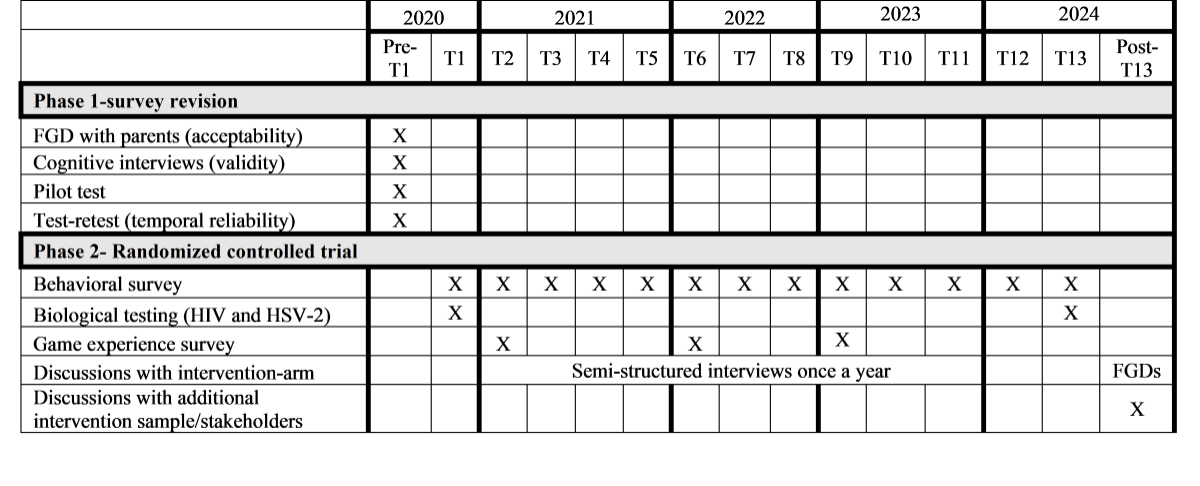
Schedule of study activities. FGD: focus group discussion; HSV-2: herpes simplex virus, type 2.

They were instructed to engage in a minimum of 10 hours of game play over each game play period during the long school holidays in December of the first year of the study and in the 2 subsequent years. However, due to COVID-19 disruptions to the school calendar, participants had a December 2020 game play period that will be followed up by a March 2022 game play period, as only these holidays provided a long enough duration for meaningful intervention exposure. The game automatically collects data on participants’ in-game behavior, logging time-stamped records of all participant interaction with the app. These will subsequently be analyzed to assess, for example, time spent playing, scores on knowledge-based mini-games, choices made in the narrative game, and components to which the player was exposed. Adolescent participants enrolled in the control arm receive *Brainilis* loaded on a phone identical to that used for the intervention-arm participants at the same time the intervention game is made available to that study arm. Provision of a control game to the nonintervention participants is intended to ensure that any intervention effect was due to the content of the intervention rather than to a phone and game more generally. All enrolled adolescents will complete 13 behavioral surveys over the 45-month period via ODK. Following each game play period, they will also complete a game experience survey (n=3) and indicate which game player profiles are theirs and which were created by family members and friends, for analytical purposes. A schedule of activities is presented in Figure 1. Similarly, following disruption to the school calendar and subsequent reduction of most school holidays to about 1 week until 2023, there was a need to shift resources to accommodate administration of surveys for all participants within the 1-week window.

All study participants completed baseline, and will complete endline, HIV and HSV-2 testing. These procedures are carried out by trained nursing staff at the participants’ nearest health facility of their choice. Pre- and posttest HIV counselling is provided as per national guidelines. HIV testing follows a modified national algorithm, involving parallel approved antibody-based rapid test kits at the health facilities, and is confirmed (for discordant results) by HIV DNA polymerase chain reaction at a centralized laboratory. The rationale for the modified HIV-testing algorithm is to reduce chances of false-positive test results, given the relatively low HIV prevalence in the target population, and the attendant risk of causing needless distress to adolescent participants. HSV-2 testing is conducted using HSV-2 enzyme-linked immunosorbent assay (ELISA) kits at a centralized laboratory. Study staff followed up with the participant and testing facility to ensure linkage to care for those who were newly diagnosed, who were contacted 2 weeks after testing for workup to initiate antiretroviral therapy if this could not be done immediately. Participants were retained for the study regardless of their baseline biological test results.

### Staff Training and Information Materials

All research personnel completed ethics training and underwent an intensive protocol training before each round of scheduled activities. They reviewed data collection tools, had access to a phone loaded with *Tumaini* and *Brainilis* ahead of the recruitment to familiarize themselves with the games, and reviewed study-specific data collection procedures and a manual of study standard operating procedures. All research team members involved in qualitative research data collection had previous experience, including relevant training during the feasibility study of the intervention. The site management team had previous training in the provision of adolescent-friendly services for sexual and reproductive health research. Targeted health facility nursing staff were already trained on biospecimen collection and adolescent-friendly HIV counselling and testing procedures. The study team includes individuals trained in counselling who assist with identifying emotional distress and refer participants for gender-based violence support as needed.

The study team developed informational handouts based on feedback and experience from the pilot research and from staff training. These were provided to the recruitment and enrollment staff, enabling them to respond to parents’ and adolescents’ questions and concerns about the study and the game in a uniform way and accurately represent the goals of the study.

Due to the ongoing pandemic, all study personnel also received training on COVID-19–related protocols for personal and participant health and safety, including the proper use of personal protective equipment (PPE). Key study personnel were already familiar with Zoom teleconferencing software. Staff training was conducted on recognition and documentation of signs and symptoms related to COVID-19, including the need for self-isolation and access to appropriate services as recommended by the local Ministry of Health in case of an exposure or confirmed case of COVID-19.

### Phone Setup

*Tumaini* and *Brainilis* were downloaded and programmed into the intervention and control phones, respectively. Similar to the setup used during the feasibility test, all other phone functions were disabled using a parental control app. The same phone will be issued to the same participant during each game play period in order to allow the participant to resume prior game play, if desired. Where phones are lost or cannot be restored, a replacement will be logged and issued. The phone identification number and participant identification number are linked in a separate phone log, which will be used as reference for every phone allocation visit and to match in-game data from *Tumaini* log files to participants’ other study data in the LabKey data management platform. To complement data collected through the ODK behavioral surveys, intervention-arm players’ in-game log files (paradata) will be downloaded upon return of the phones each year. These files contain time-stamped details of player interaction with the game, which will be used to examine exposure to the intervention.

### Qualitative Data Collection and Postintervention Procedures

The intervention efficacy activities are being complemented by qualitative monitoring of a sample of the intervention arm. A cohort of adolescent participants from this study arm and a sample of parents of adolescent participants, including 6 parent-child dyads, have been selected to take part in semi-structured interviews (SSIs) once a year during the trial and in FGDs at the end of the trial. These 28 individuals were sampled to represent different SES profiles and types of game players. SES was based on reported food insecurity and house materials on the baseline behavioral survey, while player type was drawn from the behavioral survey (experience with video games) and the game experience survey administered after the first game play period (self-reported time spent on *Tumaini,* degree of completion of the game, and engagement with others about *Tumaini*). In addition to annual SSIs, this cohort will also be invited to share insights into their experiences throughout the study during post-endline FGDs.

A further 8 FGDs will be convened after endline, 4 for additional intervention arm participants and 4 for additional parents, with participants being selected in a way similar to the SSI cohort to represent a range of demographic and intervention-user profiles. Other stakeholders from the community (n=32; eg, teachers, healthcare providers, community leaders) will be invited to participate in endline SSIs or FGDs. These SSIs and FGDs will elicit feedback on the game and study experience that will inform future game dissemination and scale-up and development of future interventions and trials.

### Other COVID-19 Adaptations

In order to minimize risks related to COVID-19, study activities are being conducted remotely (eg, video conferencing, phone calls) to the extent possible. As remote data collection relies on participants having a phone, tablet, or computer with internet access in their households, it entails SES bias and hence is only deemed feasible where such bias does not threaten the scientific integrity of the study.

In cases where study activities cannot be conducted remotely, these are conducted at a distance of 2 to 3 meters (about 6-10 feet) from study staff or other participants. Prior to all in-person contact, participants and study staff are screened for symptoms and undergo temperature screenings; these data will not be analyzed as part of this study. Physically distanced study activities (eg, SSIs, informational sessions, consent or assent) involving verbal interaction take place outdoors to the extent possible, and both staff and participants wear masks. If physical distancing cannot be maintained (eg, for blood collection), appropriate PPE (masks and gloves) is used. If they take place in dedicated spaces at KEMRI or community-based organization offices, participants are scheduled to avoid proximity and extend waiting time, and the research area is disinfected between participants. Where electronic devices are handed to participants for survey data collection or intervention delivery, these devices are disinfected prior to distribution and after collection.

So far, all study procedures have been conducted in person following local infection prevention measures, with agreement from the study participants. Options to conduct the procedures remotely remain available to study staff and participants should they be needed.

### Safety Monitoring

An independent safety monitor (ISM), appointed for this study, was selected based on prior experience with clinical trials, HIV behavioral interventions, adolescent sexual health, and experience working in Kenya. The ISM’s eligibility and lack of conflict of interest were independently confirmed. The ISM reviews study materials including participant safety data and overall study conduct as specified in the protocol. The ISM addresses issues of research participant protection, by examining over time the safety data from the study in order to evaluate safety findings and trends and make recommendations concerning continuation, termination, or other modification of the study based on the observed beneficial or adverse effects and social harms associated with the intervention. In addition, the ISM reviews the general progress and conduct of the study regularly and assists in resolving any problems that may arise.

### Sample Size

At age 13 years, according to the most recent available data [45], in Kenya, 4.2% of female and 12.8% of male adolescents have reached sexual debut; by age 17, these figures rise to 40.9% and 42.3% respectively. Condom use at first sex is reported to be 22.8%, 43.3%, and 67.2% for those having sex by age 13 years, before 15 years of age, and after 15 years of age, respectively [45]. Based on these estimates and outcomes of other interventions targeting similar behavior in this age group [14,16], we calculated sample sizes based on a primary binary outcome of “risk” group (experienced sexual debut during the study period without condom use at first sex) versus “low-risk” group (not yet experienced sexual debut by the end of the study or used a condom at sexual debut during the study period). Targeting 1:1 randomization into the 2 study arms, we aimed to consent and enroll a total of 1000 participants. This would be sufficient for 80% power to detect a difference between 18% of control-arm participants “and 11% of intervention-arm participants in the “risk” group for our binary outcome at the α=.05 level, accounting for 25% potential loss to follow-up. This sample size also allows for stratification of participants by HIV status at baseline, when we anticipate around 10 participants to test positive, based on local HIV prevalence among this age group.

### Statistical Analysis

The distribution of the primary binary “risk” outcome of this study will be assessed via standard methods for comparing proportions and related 95% CIs. This will be followed by logistic regression modeling to account for covariates, to be determined after analysis of baseline demographic and anticipated confounder data. The crude mean and median age at first sex will be ascertained for all participants who experience their debut over the course of the study, augmenting this analysis to account for gender and other covariates using parametric or semiparametric regression models as appropriate. As with the primary outcome, the binary response of condom use at first sex among those experiencing debut during the study will be analyzed via logistic regression. To determine whether the game has the potential to reduce HSV-2 or HIV infection, incidence rates over the course of the study of HSV-2 and HIV among uninfected participants will also be compared by exposure to intervention using Poisson and Cox regression methods.

To determine whether the game-based intervention influences behavioral mediators (knowledge, attitudes, behavioral intentions, and related self-efficacy) of early and unprotected sex, crude and adjusted associations between behavioral mediators and exposure to the intervention will be explored using multivariable regression methods appropriate to each potential mediator, following stipulated principles of mediation analysis [46]. Using baseline behavioral survey data, factor analyses will be conducted to identify theoretical and thematic scales. Survey data will be analyzed both as individual items and as composite scores for these scales. Analyses of outcome data, game experience survey data, and the paradata from *Tumaini* players’ smartphones will be used to assess which game components and theoretical constructs mediated intervention effects [46,47].

## Results

**Figure 2:**
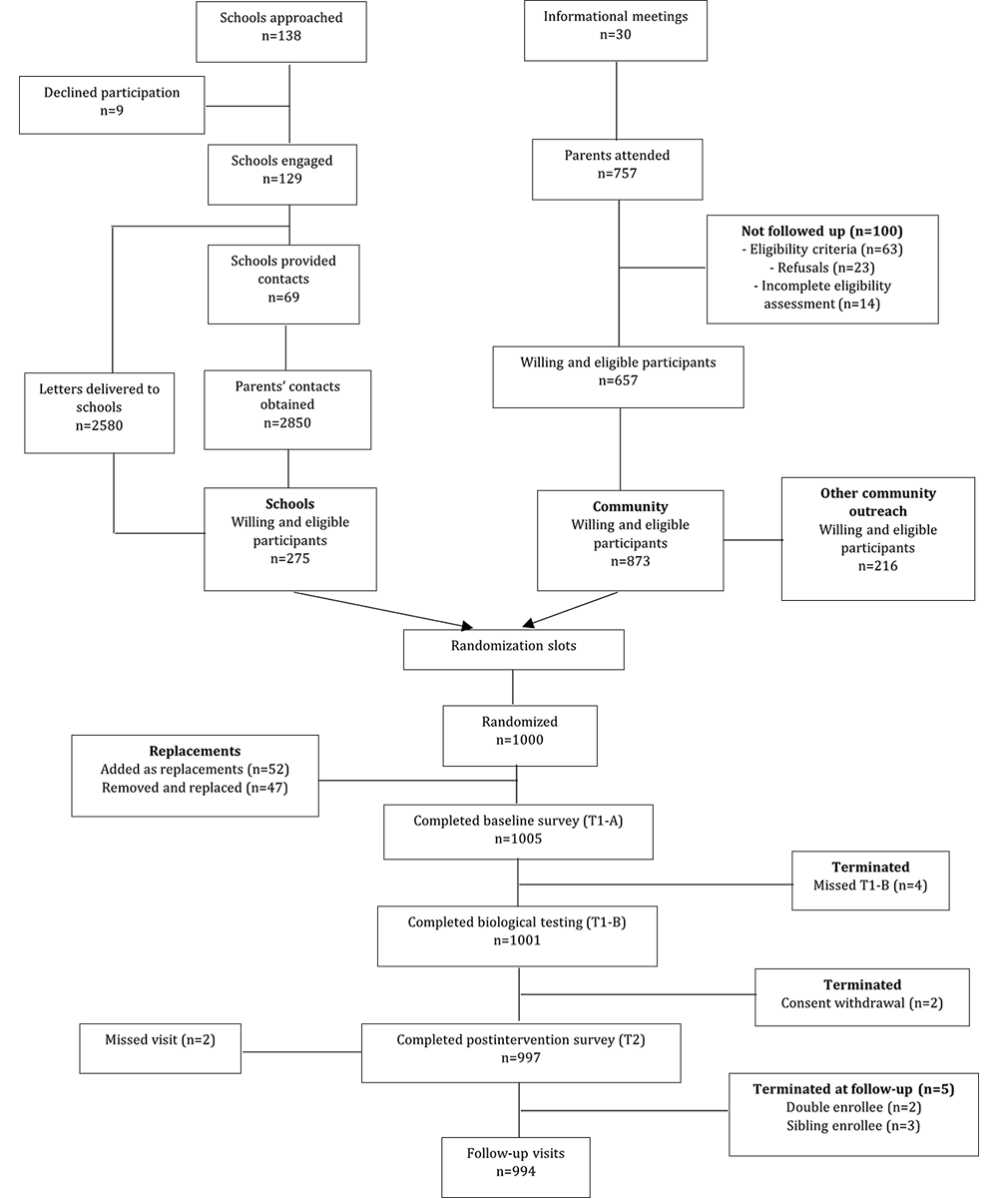
Trial flow diagram.

Recruitment, consenting, and enrollment were carried out between October 1, 2020 and December 3, 2020. In line with our COVID-modified protocol and with schools closed, a total of 138 primary schools were approached within Kisumu County, either directly to distribute recruitment letters or to provide contact details of parents with children in grade 7 (Figure 2). Recruitment letters were randomly distributed to willing schools, but we did not track how many letters were subsequently issued by schools to potential participants. From contact details obtained from schools, the majority of the potential participants were ineligible due to those adolescents not being in grade 7 or within the target age range. There was generally very low yield from the school-based activities due to school closure, which were deemed inefficient and stopped midway through the recruitment period; hence, some contacts were not followed up on.

Within the same period, 30 informational meetings were held in the community, with 757 parents attending. Of these, a small fraction was ineligible due to their child being older than 14 years, in grade 8, or failing to pass the English proficiency test for the study. The main reasons for parental refusal included concerns with blood draws, concerns with the adolescent being issued a phone, fear of HIV testing, concern with adolescent safety, time constraints associated with study visits, and unwillingness to participate in research. The majority of the participants in this study was enrolled from community meetings.

As noted previously, recruitment randomization slots for 1000 individuals were generated and used to ensure distribution of the sample by gender, age, and residential area prior to randomization by gender. There was a time lag between being recruited and administration of the baseline survey for some participants; hence, a number of eligible and willing adolescents could not be traced for enrollment or had moved out of the study area. After sampling from the full pool of interested and eligible individuals, 148 willing and eligible adolescents who were not randomized served as a pool for replacement for those who could not initiate study activities. The randomization slot characteristics were used to identify suitable replacements from the pool. During this replacement period, some participants who were identified as needing to be replaced ended up presenting themselves for a baseline survey appointment; hence, the study ended up with 1005 enrolled individuals completing the baseline survey. There were 4 participants who completed the baseline survey only and not the biological testing; they were removed from the study. Baseline behavioral surveys were conducted between November 17, 2020 and December 8, 2020, and biological testing was conducted between November 25, 2020 and December 12, 2020. Study phones with either the intervention or control game were distributed after completion of the biological tests; participants were in possession of the phones for a median period of 37 (IQR 34-40) days.

Postintervention surveys were conducted between January 2, 2021 and January 27, 2021, at which point the study team discovered a participant who was enrolled with 2 different study identification numbers resulting from presenting themselves twice and 3 different cases of siblings living within the same household enrolled in contravention of the protocol. The double enrollee was terminated from the study. In cases where the siblings were in different study arms, the intervention arm participant was retained. In cases where they were both in the same arm, the study criteria for selecting a sibling at recruitment was applied—the first participant based on alphabetical order of their first names was retained. After elimination of individuals later found to be ineligible for these reasons, the study had 1001 participants. The total number who completed baseline and will be included in baseline analyses is 996.

Demographic characteristics of these 996 participants who completed all baseline activities, were allocated to the 2 study arms, and received a phone loaded with either the intervention or control game were evaluated (Table 1). This analysis excludes the double enrollee and 3 siblings who were terminated. The participant sample was 50.1% (499/996) female and had a mean age of 14.0 (SD 0.6) years. At baseline, participants were almost universally enrolled in school, with the majority reporting either grade 6 or 7 as the highest grade they had completed. SES, using housing materials as proxy, was estimated to be almost evenly split between “higher” (507/992, 51.1%) and “lower” SES (485/992, 48.9%), with 4 participants not providing this information. On the combined measure of food insecurity (ie, household hunger either during the day or night or overnight), more than one-half (530/996, 53.2%) of participants reported no food insecurity (“never” responses on both questions), while 33 (33/996, 3.3%) reported high levels thereof (“sometimes” or “often” responses on both questions). Close to 90% (883/996, 88.6%) of participants reported living with at least one parent, with the others, for the most part, in the care of grandparents or other adults, including aunts, uncles, and other guardians. The majority (892/977, 91.3%) of participants identified as Christian (Catholic, Protestant/Anglican, Seventh Day Adventist), while a small number identified their religion as Muslim (31/977, 3.2%) or another local religion (21/977, 2.1%) or reported not being part of a religious group (33/977, 3.4%). Religiosity is high among participants, with 78.0% (777/996) reporting attending services once a week. Household technology access is relatively high, with only 17.9% (178/996) of participants reporting no household smartphone ownership, and 37 (37/996, 3.7%) indicating that they themselves owned a smartphone. We also assessed prebaseline levels of engagement with video games. Although 22.8% (227/996) reported never playing video games, 114 participants (114/996, 11.4%) indicated that they often play. Analysis by arm has not commenced as the study is ongoing; these demographics are therefore presented in aggregate rather than by study arm.

**Table 1:** Participant demographics (n=996) at baseline.

Characteristics		Results
**Sex, n (%)**		
	Female	499 (50.1)
	Male	497 (49.9)
Age (years), mean (SD)		14.0 (0.6)
Attending school (yes), n (%)		992 (99.6)
**Highest school grade completed, n (%)**		
	Grade 5	43 (4.3)
	Grade 6	630 (63.3)
	Grade 7	280 (28.1)
	Grade 8	43 (4.3)
**Socioeconomic status^a^, n (%)**		
	High	507 (51.1)
	Lower	398 (48.9)
	Missing	4 (—)
**Household food insecurity^b^, n (%)**		
	None	530 (53.2)
	Low	287 (28.8)
	Medium	146 (14.7)
	High	33 (3.3)
**Living situation, n (%)**		
	Both parents	547 (54.9)
	Mother only	276 (27.7)
	Father only	60 (6.0)
	Grandparents	72 (7.2)
	Orphanage or children’s home	3 (0.3)
	Other living situation	38 (3.8)
**Religion, n (%)**		
	Catholic	356 (36.4)
	Protestant/Anglican	293 (30.0)
	Seventh Day Adventist	243 (24.9)
	Muslim	31 (3.2)
	Other local religion	21 (2.1)
	No religion	33 (3.4)
	Missing	19 (—)
**Attendance at religious services, n (%)**		
	Once per week	777 (78.0)
	1-2 times per month	77 (7.7)
	A few times per year	83 (8.3)
	Once per year or less	22 (2.2)
	Never	37 (3.7)
Household access to smartphone, n (%)		818 (82.1)
**Frequency of video game play, n (%)**		
	Never	227 (22.8)
	Rarely	365 (36.7)
	Sometimes	290 (29.1)
	Often	114 (11.4)

^a^Determined by materials used to construct their homes.

^b^Based on scoring index developed from reported number of days household went hungry or slept hungry.

## Discussion

### Study Overview

Based on findings from our pilot study of *Tumaini*, this study aims to determine the efficacy of the intervention in delaying sexual debut and increasing condom use at first sex. This study will also determine whether the game-based intervention influences behavioral mediators (knowledge, attitudes, behavioral intentions, and related self-efficacy) of early and unprotected sex and which particular game components and theoretical constructs mediate the desired effects. This study responds to the need for an increased number of behavioral and structural HIV intervention options appropriate for adolescents [48], as adolescents and young people require tailored approaches to meet their needs. The game will also collect data to inform potential implementation on a wider scale, if warranted.

The COVID-19 pandemic has led to substantial changes in routine daily activities and social interactions. Through these and other challenges, the pandemic has affected implementation of ongoing and planned research across the globe. There is a call for research teams to get creative about ways of reaching, engaging, and reimbursing study participants [49] and, in doing so, follow the guidelines provided by local authorities and ethical review committees. The study local ethics and research committee provided guidelines that aim to protect trial integrity and study subjects [50], which informed the proposed changes to the protocol. Researchers have had to identify activities that do not place the study participants at increased risk of COVID-19 while maintaining study rigor, amid debates about how implementing research amid the COVID-19 pandemic may affect the balance between participant risks and benefits [51]. Physical distancing to protect participant and researcher safety has been one of the major concerns with research implementation at this time. For this study, we were able to offer remote consenting; virtual interview options using Zoom software or phone calls; and, whenever physical contact was inevitable, maintaining recommended social distancing, enforced use of appropriate PPE and a shift in planned activities to accommodate the availability of the target population. Online platforms have been shown to be viable options for conducting research during the pandemic [44], with the need for the researcher to be aware of the inherent potential biases [52] and of the limitations of lack of nonverbal cues and privacy and access issues [53]. In our case, despite securing ethics approval, anticipating a need for remote activities, and taking measures to minimize any biases associated with technology access, no participant was consented remotely, nor did any study activities occur via teleconferencing. This is largely because local meetings were permitted with appropriate social distancing and face coverings and were preferred by our participants.

The pandemic has further highlighted some of the advantages of *Tumaini*’s remote delivery platform, and this study is likely to inform creative ways with which we target adolescents with HIV prevention interventions. Many of the countries in sub-Saharan Africa are adopting comprehensive sexuality education amid varying challenges, including unreceptive sociocultural norms, parental attitudes, teacher-related challenges, and economic factors [54,55]. There is considerable interest in mobile health (mHealth) to support the delivery of HIV care and prevention services in the general population, as this offers an opportunity to provide such education at scale and low marginal cost [56]. However, very few mHealth interventions are specifically targeting adolescents with customized messages. If found to be efficacious, *Tumaini* will provide an alternative, appropriate, acceptable, and contextualized mode of passing HIV prevention messages and skills building to adolescents within sub-Saharan Africa.

### Limitations

The study design has inherent limitations, which have been compounded by COVID-19–necessitated modifications and implications. First, there is a risk that participants in the intervention arm may discuss or even share the *Tumaini* app with participants in the control arm. To reduce the risk of contamination, study visits will be scheduled such that participants from the 2 arms do not mix during data collection activities. Second, survey activities and game play periods were planned to coincide with normal school holidays, which would be at least 3 weeks, with the game play periods planned during the year-end long holiday period, usually lasting around 6 weeks. With the shift in the academic calendar in Kenya because of forced COVID-19–related school closure, the holidays were subsequently shortened to only 1 week in most cases. This results in a shorter than desired period to conduct the surveys and game play periods. Resulting changes to game play periods may require that the study implement longer than 1-year intervals between subsequent game play periods, with the risk of affecting recall of game contents and key messages and skills in the interim. Third, there are limitations with using self-reported outcomes for condom use and sexual debut. We are supplementing self-report data with biological measures of HIV and HSV-2 at baseline and endline; however, the study is not powered to detect differences in these biological outcomes due to relatively low rates of HIV or HSV-2 in this age group. Finally, there is an inherent threat of loss-to-follow-up over the 4 years of the study as the adolescents progressively transition through the school system or migrate outside the study area. In addition to accounting for the potential of up to 25%loss-to-follow-up in our sample size calculations, we plan to have annual parental engagement activities to keep parents engaged and inform them of upcoming study activities for the year ahead. Such engagement activities are likely to inform us of planned migration out of the study area and encourage parents to facilitate continued adolescent participation in the study.

### Conclusions

This ongoing research demonstrates that, with appropriate revisions to planned activities, incorporation of lessons learned from our previous pilot study, and compliance with regulatory guidelines, recruitment and conduct of research procedures are possible against the background of a global pandemic. This study identified and recruited adolescents for an efficacy trial to evaluate a smartphone game–based intervention aiming to delay sexual debut and increase condom use at first sex. If the trial demonstrates efficacy, it will provide an alternative means of delivering age-appropriate education to adolescents on safer sex, HIV prevention, and effective life skills. It would also identify implementation challenges and how to overcome them in a potential wider scale rollout.

## Abbreviations

CAB: community advisory board
ELISA: enzyme-linked immunosorbent assay
FGD: focus group discussion
HSV: herpes simplex virus
IRB: institutional review board
ISM: independent safety monitor
KEMRI: Kenya Medical Research Institute
mHealth: mobile health
ODK: Open Data Kit
PPE: personal protective equipment
RCT: randomized controlled trial
SERU: Scientific and Ethics Review Unit
SES: socioeconomic status
SSI: semistructured interviews
WHO: World Health Organization
